# Find the Hidden Object. Understanding Play in Psychological Assessments

**DOI:** 10.3389/fpsyg.2017.00323

**Published:** 2017-03-24

**Authors:** Alessandra Fasulo, Janhavi Shukla, Stephanie Bennett

**Affiliations:** ^1^Department of Psychology, University of PortsmouthPortsmouth, UK; ^2^Autism InterventionistMumbai, India; ^3^Institute of Criminal Justice Studies, University of PortsmouthPortsmouth, UK

**Keywords:** play, psychological assessments, down syndrome, conversation analysis, learning disability

## Abstract

Standardized psychological assessments are extensively used by practitioners to determine rate and level of development in different domains of ability in both typical and atypical children. The younger the children, the more likely the trials will resemble play activities. However, mode of administration, timing and use of objects involved are constrained. The purpose of this study is to explore what kind of play is play in psychological assessments, what are the expectations about children's performance and what are the abilities supporting the test activities. Conversation Analysis (CA) was applied to the videorecording of an interaction between a child and a practitioner during the administration of the Bayley Scale of Infant and Toddler Development, III edition. The analysis focuses on a 2′07″ long sequence relative to the administration of the test item “Find the hidden object” to a 23 months old child with Down syndrome. The analysis of the sequence shows that the assessor promotes the child's engagement by couching the actions required to administer the item in utterances with marked child-directed features. The analysis also shows that the objects constituting the test item did not suggest to the child a unique course of action, leading to the assessor's modeling of the successful sequence. We argue that when a play frame is activated by an interactional partner, the relational aspect of the activity is foregrounded and the co-player becomes a source of cues for ways in which playing can develop. We discuss the assessment interaction as orienting the child toward a right-or-wrong interpretation, leaving the realm of play, which is inherently exploratory and inventive, to enter that of instructional activities. Finally, we argue that the sequential analysis of the interaction and of the mutual sense-making procedures that partners put in place during the administration of an assessment could be used in the design and evaluation of tests for a finer understanding of the abilities involved.

## Introduction

This paper looks at play within standardized psychological assessments. It analyses in detail the administration of the test item “Find the hidden object” of the Bayley Scale for Infant and Toddler Development, 3rd edition, to a 2 year old child. The scale is designed to determine developmental delay; the child observed has Down syndrome.

The study will explore what kind of play is play in a psychological assessment, what are the embedded expectations about children's performance and what is the interactional infrastructure supporting the assessment activities.

The study is conducted within an ethnomethodological framework and considers the full ecology of the activity under scrutiny, including the multimodal components of communication and the material features of the setting.

## Assessing different children

Assessing the nature and extent of children's difficulties is key to a number of highly consequential decisions for children and their families: whether they need speech and language therapy or learning support, whether they can be included in mainstream schools, whether parents and teachers need to be involved in the children's care.

Psychological assessments vary from standardized tests to less structured forms of evaluation, such as interviews or observation of free play. The latter types are based on specific behavioral indicators but also on more holistic perceptions of the assessor about a child's abilities, reactivity, and social and emotional adjustment. Standardized assessments, on the other hand, have the advantage of giving quantified descriptions of a child's level of (dis) ability that are more easily communicable and transferable, as well as allowing to measure change at different time points.

Some assessments are designed for testing particular deficits in specific populations, such as the False Belief Test that examines Theory of Mind in children with Autism (Baron-Cohen et al., 1985), whereas others, like the Bayley we examine here, measure a broader umbrella of abilities and is administered to children with very different types of difficulties or conditions. This second type of test positions the child on a variety of dimensions measured through the scoring they achieve on different subscales. Very often test-items included in the sub-scales do not descend from clearly specified developmental theories, or the experiments that may have been carried out to confirm those; instead, like in the Bayley, they may have been assembled from previous tests, and rearranged for order and features of trials. These tests therefore rely on an eclectic theorization, as well as on general ideas about the way children function, to inform the choice of particular test-objects and trial administration techniques.

The robustness of standardized assessments lies in the procedures aimed to establish their validity. Such procedures include administering them to large samples of individuals and correlating scores with other existing scales. These are accepted procedures in psychology, but they also means that the world of tests lives in and of itself, being able to produce new measuring tools but progressively losing connection with the research around determinate clinical conditions (Millon, 1987; Cicchetti, 1994).

A recurrent observation about standardized assessments is that full standardization is very difficult to achieve (Marlaire and Maynard, 1990; Antaki et al., 2002; Maynard et al., 2002). Assessments are administered in and through interactions; human interaction, in its verbal and non-verbal components, is organized to its more minute details and comprises largely automatic and habitual communicative patterns (Sacks, 1992). The behavior of the tester is unlikely to fully resist acts suggested by the interactional organization despite the test's manual recommending it. Test administrators, for example, have been shown to give feedback, and also different feedback after success or failure, when it is not supposed to happen. This may impact the motivation of the person under examination, and also induce inferences about right and wrong answers, causing “learning within the task” (Maynard, 2005) and uncontrollable effects on the overall performance.

The interactional organization can impinge on tests in other ways, independently from the violation of the test rules. For example, if a question is asked twice in ordinary conversation, speakers understand it as a request for repair, namely that the first answer needs to be in some way amended because it was not right or had not been understood or heard properly; a person under test requested to perform the same behavior twice (as many test do to confirm that a skill is actually possessed) may instead do something different, under the interpretation of having got it wrong the first time. Maynard and Marlaire (1992) refer to these occurrences as part of the “interactional substrate” of assessments, and notice the scarce attention they are accorded to within the world of test design and use.

Our study falls within an area of research in Conversation Analysis (CA), substantially shaped by the work of Maynard and Marlaire cited above, that examines the interaction occurring in the course of established assessment practices in clinical work. Research in this area focuses on how participants cooperate in the achievement of the assessments' outcomes and identifies the sense-making procedures made relevant there and then by participants for each other. Interactional studies of assessment, while at times highlighting the limitations of these tools, do not aim to undermine them but rather to provide a broader understanding of how they function, for both interactionist researcher and practitioners to build on to. Following the terminology used in Hasson and Botting (2010), Muskett et al. (2012) propose that “static” standardized assessments can be complemented by “dynamic” ones that take into account the interactional vicissitudes of the test administration, highlighting competences that the fixed scoring systems would not pick up. As recalled elsewhere (Fasulo, 2015), a rounder evaluation which would engage the child and also gather information from other sources was a default procedure in the early decades of psychological testing, even for the IQ test, so integrating different evaluations is not necessarily disruptive of the ethos of psychological assessments.

The present study looks at a test that, being addressed to young children, is designed as a series of play activities. The analysis will thus attend to the way a play framework is implemented through the test procedures, both those prescribed to assessors and those spontaneously mobilized to carry out the activities.

## What is play for young children?

Definitions of play abound and are constantly updated, as none can exhaust the infinite variety this activity can embody. Classic and broad definitions include lack of immediate utility (Huizinga, 1992/1938) and voluntary participation (Caillois, 2001), although there may be exceptions to those as well (Sicart, 2014).

Benefits of play have been recognized across different domains, developmental and psychological in general. The benefits are linked to different characteristics of play. For children, they are seen to stem from play including both structure and improvisation (Sawyer, 1997), allowing children to be creative and elaborate their experience along predefined routes. Perspective-taking is notoriously one of the most important functions GH Mead (1934) saw in playing games, i.e., structured social event built around a set of rules and roles; through playing games, he argued, children understand that social roles are positional and learn to imagine the world from another person-role perspective.

Some see a socialization benefit in the rehearsal and familiarization with activities of the adult world (Lancy, 1996); this kind of benefit, it is also argued, is not unique to humans. Animal play has been recognized many functions, including that of exercising flexibility rather than learning repetitive patterns of behavior (Bekoff and Byers, 1998). Generally, following Bateson's insight about play as happening within a communicative *frame* (Bateson, 1956), it is rather safe to say that play develops tools for the fine layering and articulation of meaning, bestowing on intra- and interspecific communication a wider range of possibilities^1^.

Rules are central in play as they are constitutive of the alternative sphere of reality play lives in. However, first, in the classic distinction between play and games it pertains only to the latter to be *dependant* on rules for their existence; secondly, even within games, players are seen to bend, or recreate rules and develop different games from within the original ones. True play, in other words, has always the power to reinvent itself (Sicart, 2014). A fundamental characteristic of play is in fact *appropriation* (Henricks, 2006), i.e., the capacity to invest of new meaning any setting or object at hand and make it become what the players wish it to be. The appropriative nature of play makes game design or play scripts subservient to playing itself: those can support and extend players' imaginative capabilities, but play can happen without pre-designed artifacts or can put them to different use than they were originally designed for.

The characteristics of play described above are in various degrees related to the fact that play is an instrument for self-creation and self-expression through shared semiotic means, in this similar to language (Sutton-Smith, 1997). Without the freedom to interpret a play situation, the essence of play would be gone, although the situation can retain formal play features. In the same vein, toys can cue certain actions or cue play as such, but they are not to be seen as imposing limits to play; as Sicart (2014: 44) argues, toys can be used as a starting point to *filtering* the reality around them to create an apt play environment. The toys' own physicality, on the other hand, is crucial in orienting the shape and experience of play.

It is difficult to ascertain to what extent young children, especially if pre-verbal or with low verbal capabilities, distinguish play from other activities they are involved in. Observations of children in the first year of life show that caregivers and children participate in play routines - such as nursery rhymes, interactive songs and the like–that engage simultaneously multiple senses and modalities (for example associating singing with touching and moving the body) and have recognizable trajectories (Fantasia et al., 2014); at the same time, many functional activities, such as feeding (Costantini, 2015) or nappy changing (Nomikou et al., 2016) are suffused with play and present similar characteristics of regularity and multimodality.

Objects enter the world of children since the early days, initially designed to stimulate children in rather passive ways, with their sound and tactile properties, –like with like infants' books (Rossmanith et al., 2014)–then increasingly imbued with “narrative programmes” (Greimas and Courtés, 1982) that comprise a diversified range of actions. Objects that children can engage with are not limited to toys: sheets, clothes, care products, feeding accessories and the like can also be manipulated and explored. Non-functional, explorative manipulation of objects is a spontaneous activity in children and can be picked up by caregivers to extend the child's repertoires of actions and favor participation in mediated interactions (van Oers, 1998).

The research on play summarized above will inform our analysis of the administration of the Bayley's item “Find the hidden object,” particularly as concerns explorative aspects of play and the role of objects in the activity.

## Children with down syndrome as interactants

In the following we will briefly summarize only the characteristics of children with Down syndrome that are relevant for their ability to partake in social interaction.

Children with Down syndrome tend to be delayed in language development, for a combination of reasons, including hearing deficits due to congestion of the middle ear (“glue ear”) and issues with working memory that may make long sentences difficult to deal with. Their receptive vocabulary is closer to typical levels than their productive one: they often present limited syntactic abilities, so their utterances may often be incomplete; finally they may have difficulties in articulation due to morphology of the mouth (Chapman and Hesketh, 2000).

Conversational skills in children with Down syndrome are higher than those of children with the same level of expressive linguistic development; the quality of their social relationship is similar to that of typical children and higher on average than observed in children with Williams syndrome or Specific Language Impairment (Laws and Bishop, 2004). As the children grow older, the linguistic performance can be lower than the cognitive ability would allow, suggesting the effect of restricted opportunities for interactions in the first years compared to typical children (Gullberg et al., 2008).

Overall, children with Down syndrome appear well equipped to engage in prolonged social interactions, provided that the speech addressed to them is not overly complex and the interlocutors learn to overcome occasional disfluencies in their speech.

## The Bayley-III test and the item “find the hidden object”

The first Bayley Scales of Infant and Toddler Development was designed by Nancy Bayley in 1969, on the basis of several experiments she herself conducted in the early 60s. Two more editions were since published, one in 1993 and one in 2005 (Bayley, 2005); the last edition includes more sub-tests with the aim of distinguishing more clearly between cognitive, linguistic and social-emotional abilities (Albers and Grieve, 2007; Maccow, 2008).

The Bayley-III can assess children from 1 to 42 months and can take 30–90 min to administer, depending upon the age of the child. The main declared purposes of the Bayley-III are to identify children with developmental delay^2^ and to provide information for intervention planning; however, there is not much evidence supporting the utility of the Bayley III for intervention (Albers and Grieve, 2007). The assessment is derivative of several scales based on older and newer concepts in developmental studies and has therefore an eclectic theoretical foundation (Albers and Grieve, 2007).

The Bayley is a so called power test, i.e., one in which items are ordered according to their degree of difficulty. Children start at an age-specific point and have to pass three consecutive items on that level to go further, otherwise they are made to start again at a lower age level. The administration is stopped when the child has scored 0 in five consecutive items (Maccow, 2008). The Bayley-III comes with a thick manual containing detailed instructions, and adherence to the standardized procedures is recommended to enable use of the quantitative results of the test.

The task “Find the hidden object” is part of the Cognitive Scale, which assesses children's play skills as part of their cognitive abilities (Maccow, 2008). This test item was present in older version but used different materials, i.e., children had to find an object hidden under rather large cups. In the new version, they need to find a pink plastic bracelet under one of two pale yellow facecloths. The change was introduced because of observed difficulties in manipulating the cups.

This study, building on Shukla's (2010) finding that several children in her sample failed this task for reasons seemingly unrelated to a lack of the abilities the item is supposed to measure, apply sequential analysis according to CA procedures to one complete episode of “Find the hidden object,” with the following aims:

- To investigate the nature of play within the assessment, both as a framework for the test activities and as a set of skills measured by them;
- To identify the interactional details leading to different outcomes across repeated trials;
- To explore to what extent this kind of assessments can contribute to the understanding of individual children, or of a condition like Down syndrome more generally.

## Data and methods

The filmed data on which this study is based comes from a center for the support of children with Down syndrome and their families in the south of England. The administration of the Bayley was video-recorded as part of the standard practice of the center; for this study the selected families were contacted again for authorizing further analysis on the data.

Within a corpus of 40 children recorded doing the Bayley at two different ages (one around 12 and one around 24 months), 6 were originally selected on the basis of selected items of the MCHAT scale (Modified Checklist for Autism in Toddlers; Robins et al., 2001). This scale is compiled by parents of toddlers and asks them to report presence/absence of behaviors that may be relevant for a diagnosis of autism. Responses from 11 out of 23 questions, pertaining to range of movements and communication abilities, were used to in order to have a varied group of children for the interactional analysis (Shukla, 2010). The study led to the identification of the item “Find the hidden object” as particularly useful to illustrate the functioning of play within assessments and the interactional resources mobilized in the assessment situation.

In this paper we examine the administration of the test-item to one child, Kevin, 23 months old at the time. Like most of the studies cited in the introduction for this research area, this work uses a single-case approach in order to ground the analysis in numerous conversational episodes and throughout unfolding interactional events.

The sub-sample of MCHAT responses indicated that Kevin had difficulties in walking and hearing and did not frequently engage in active practices of joint attention.

The assessment was conducted by a woman professional. Kevin's father was also present and in this particular trial was keeping him on his lap. Assessor and parents are often visible in the recordings, but they have been purposefully cut out from the frame grabs used in this paper.

The section of the video analyzed lasts 2′07″ and includes four trials, the first three of which will be presented in the results section. The whole section was watched repeatedly and fully transcribed according to Jeffersonian conventions (Jefferson, 2004a; see Appendix for a legend of the symbols; the child's name used in the transcript is a pseudonym). Transcripts include descriptions of most gestures, expression and postural changes, as well as features of voice quality that are not captured by transcription symbols. Frame-grabs illustrating action at relevant analytical points have been added as photo-strips, each single photo referred to in the transcript as G. 1a, G.1b etc., with G indicating grab, numbers referring to the Figure which includes the grab and a, b, c indicating the specific grab in the strip.

## Results

As explained above, we will examine the first three of four trials that the administration of the item “Find the hidden object” was comprised of. By ‘trial’ we mean a cycle of activity that starts with hiding the object and ends after the child performs an action upon the object or its cover. The child did not succeed within the first two attempts, so technically he did not pass this item, but it is customary in assessments to try to achieve success anyway; the third and fourth trials see Kevin consecutively succeeding in finding the object hidden on different sides, as per the test requirements.

The child had encountered the same materials—bracelet and facecloths–in the previous round of testing, on the same day, for the lower age level; the task at that level only requires finding the bracelet under the cloth right after it is put there, whereas in the second round the cloths are swapped around before letting the child attempt to find it. Kevin had succeeded uncovering the bracelet at the first trial during the first testing round, but had failed to do so twice consecutively on opposite sides. This is why the assessor comments on this item being a “tricky one” at the beginning of the new presentation.

The extract below shows how the activity is set up. The child is sitting on his father's lap with the chest touching the edge of the empty table in front of him. He is restricted in his movements apart from the arms and head, his position maximizing his access and focus on what happens right in front of him, namely what the assessor does and what she puts on the table.

Extract 1a begins with the assessor who, after consulting the manual to check what the next test item will be, comments loudly on that “=oh this a tricky o:ne,”, apparently addressing both parent and child. She then gets up to fetch bracelet and cloths. Kevin follows her with his gaze and bangs his hands on the table, then keeps his hands flat open there. His demeanor indicates engagement with the situation and the expectation that the assessor will make something happen, most likely with objects appearing on the table as she has been doing regularly for the last 20 minutes or so.

**Table:** **Extract 1** [Figure 1]

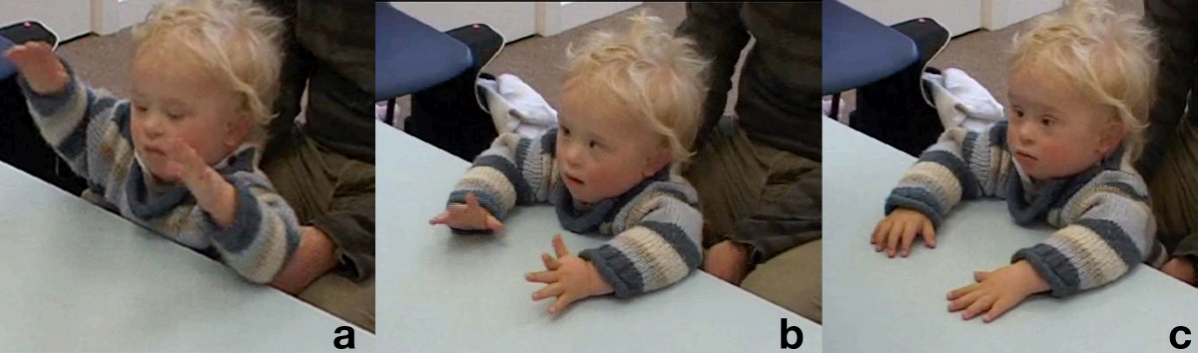		
Figure 1(a), Figure 1(b), Figure 1(c).		
		((*The assessor (A) is leafing through the manual*;
		*Kevin (K) looks at her then bangs the palms on the table*)) **G. 1a**
1	Assess:	Whe:re are we ↑no:w= ((*reading manual, K looks up*)) **G. 1b**
2		=oh this a tricky o:ne,
3		(1.5) ((*A gets up to take the material – K follows her with his gaze*))
4	Assess:	((*sits down))* <This i:s a tricky o:ne.> ((*K looks at the objects*)) **G. 1c**
5	Kevin:	Uhduhdhu:dhu,
6	Assess:	Yeah:, ((*smile voice*))

The assessor sits back at the table and repeats the sentence with more accentuated child-directed communication features, i.e., louder, more staccato and slow, with more emphasis (Baron, 1990). After this, Kevin produces a rather long vocalization (line 5) followed by the assessor's “Yeah:” (line 6) in “smile voice” (Jefferson, 2004b). Both assessor and child seem thus to be orienting to the situation as an interactional one, an object-mediated playing together, introduced by this vocal exchange. In the continuation of the sequence, in Extract 2, we can see how the play frame is sustained by the assessor throughout the delivery of the trial.

**Table:** **Extract 2** [Figures 2 and 3]

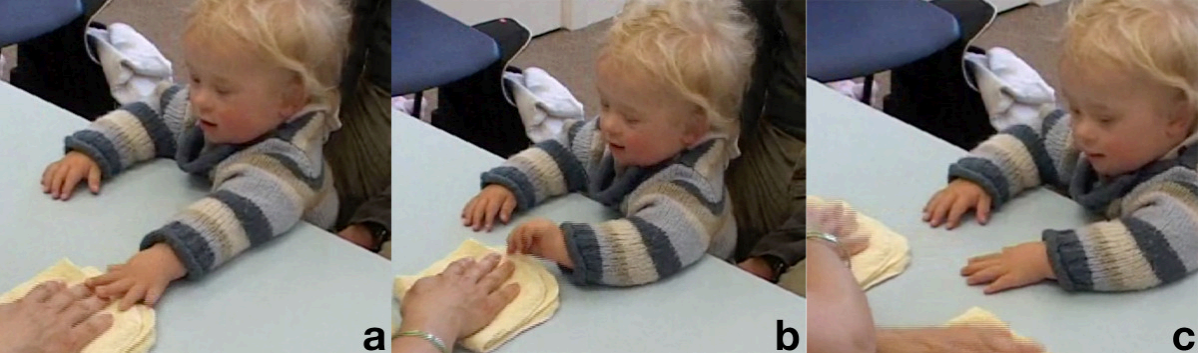		
Figure 2(a), Figure 2(b), Figure 2(c).		
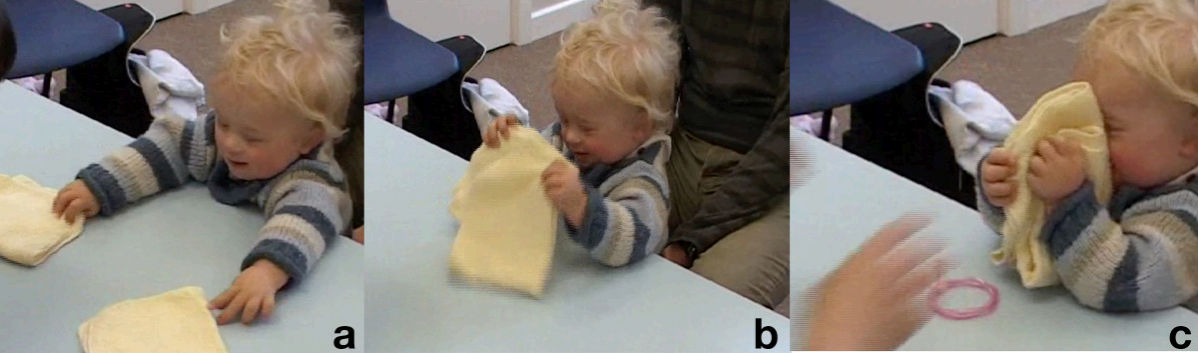		
Figure 3(a), Figure 3(b), Figure 3(c).		
7	Assess:	You watching this one again?
8		ready::?.phhhh *[*oo:ooh
9		*[*((*A places cloths on the table*))
10		(0.7) ((*A refolds one of the cloths*))
11	Assess::	*[*You watching?
12		*[*((*A holds bracelet vertical in front of K*))
13		I'm gonna hi::de the bracelet,.oh:: *[*under he:re-you w↑atchin?
14		*[*((*A slowly hides bracelet under cloth*
15		*[*((*K reaches out for the bracelet*))
16	Assess::	.Hhhh *[*w↑atchin?
17		*[*((*A presses the palms of her hand on top of the cloth*))
18		*[*((*K puts his fingers on the edge of the cloth*)) **G. 2a**
19	Kevin:	*[* Hhhe =
20	Assess:	=*[*Ohooooooo:::!
21		*[*((*A begins swapping the cloths crossing her arms*))
22		((*K, pinches and pulls at the edge of the bracelet-cloth when it passes in front of him*)), **G. 2b**
23		*A keeps the cloth down*.
24		*K's gaze stays in the middle, where the non-bracelet cloth comes into vision*)) **G. 2c**
25	Assess:	°U.h:. ((*K gazes at her*))
26	Assess:	Where is the bra:celet?
27		(.3)
28	Assess:	°Where *[*i:s it?°
29		*[*((*K reaches for both cloths*)) **G. 3a**
30	Kevin:	Hehethe:::= ((*brings both cloths toward face, deep smile*) **G. 3b**
31	Assess:	=Y:e*[*ah you want to take [both at the same t:ime,= **G. 3c**
32		*[*((*A takes cloth from K*))
33	Father:	[Hehehehehe

At the beginning of Extract 2, the assessor shows the bracelet to Kevin, with verbal and gestural highlighting (Goodwin, 2003,^3^), and he immediately reaches for it. When the assessor then covers it with the cloth and starts moving it, the child's touching and pinching the cloth looks like an attempt to keep track of it and take it from under the cloth (G. 2a,b). The rest of the sequence shows the child shifting his gaze to the hand that has crossed over to the right, then looking at the assessor when she asks him to find the bracelet, then down again at the cloths now free from the assessor's hands. At this point Kevin gleefully brings both cloths up toward his face, with a vocal comment (line 30) and pays no attention to the bracelet he has thereby uncovered. The combination of gesture and vocalization makes the picking up of the cloth an interactional move, with “response” properties with regard to the the assessor's questioning in lines 26 and 28. The assessor takes up the child's utterance with a “=Y:e*[*ah” (line 31) then comments that the child had “wanted” to do something else entirely (line 31 “you want to take [both at the same t:ime,=)^4^.

This sequence could be interpreted as showing the child's failure in keeping the focus on the bracelet and identify its position after it was moved. The analysis, however, makes at least plausible that Kevin is playing a different game here, one centered on the human interactant rather than on the bracelet. The child's attention appears mostly focused on the assessor throughout the episode: when he attempts to grasp the bracelet initially, it is after she has offered it to him. When she then rests her hands on the cloths, it is the cloths that Kevin tries to grab, her resistance to let go of it possibly making it even more playful. Finally he picks up the now free cloths vocalizing and smiling. In essence the assessor, by talking in a playful voice throughout and accompanying each small part of the trial with utterances addressed to the child, might have been creating a framework in which the child was relating primarily to her and engaging with the objects she had also been physically engaged with^5^. Furthermore, because many of the previous test items involved the child imitating what she did, Kevin might have monitored her actions in order to do the same thing again, which in this case would have been manipulating both cloths together with the two hands.

For what concerns his focus on the target object, it can be hypothesized that the soft and warm facecloths were equally interesting to him than the plastic rigid bangle; indeed, Kevin does not seem to be interested in the bracelet once its connection with the assessor is lost, but shows evident pleasure in manipulating the cloths. So, it can at least be said that the “failure” of the child to keep track of the bracelet and uncover it might be due to the very mild attraction the object exerts upon him. Whatever the case, and possibly a combination of both, namely the child might have “forgotten” the bracelet or been unable to discover its position, while also having found a new object of interest, the sequence shows that the child is engaged primarily with the human interactant and responsive to her.

Before the next trial (Extract 3), the assessor spends some time enhancing the salience that the bracelet–or the grabbing of it–has for Kevin. She offers him the bracelet, and, when he takes hold of it, she marks the action with effusive praising.

**Table:** **Extract 3** [Figures 4–6]

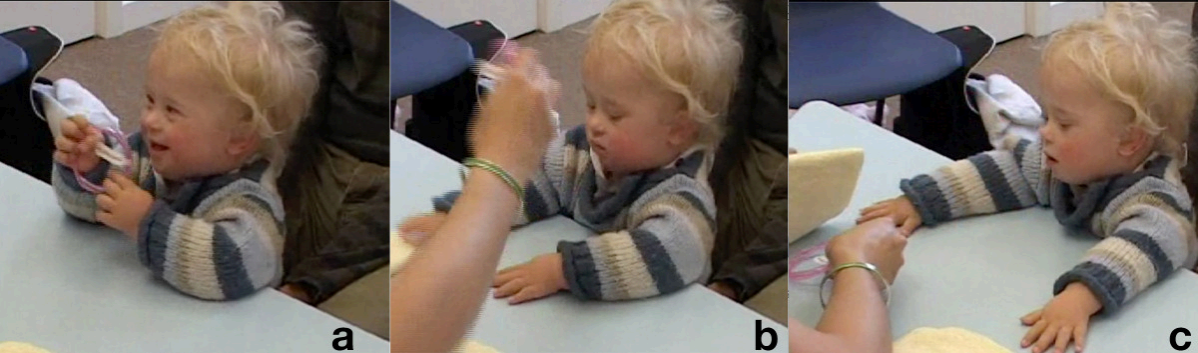		
Figure 4(a), Figure 4(b), Figure 4(c).		
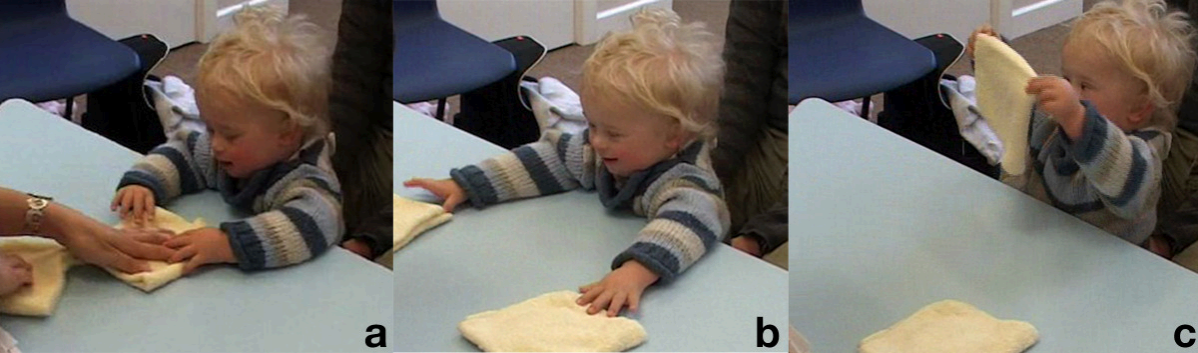		
Figure 5(a), Figure 5(b), Figure 5(c).		
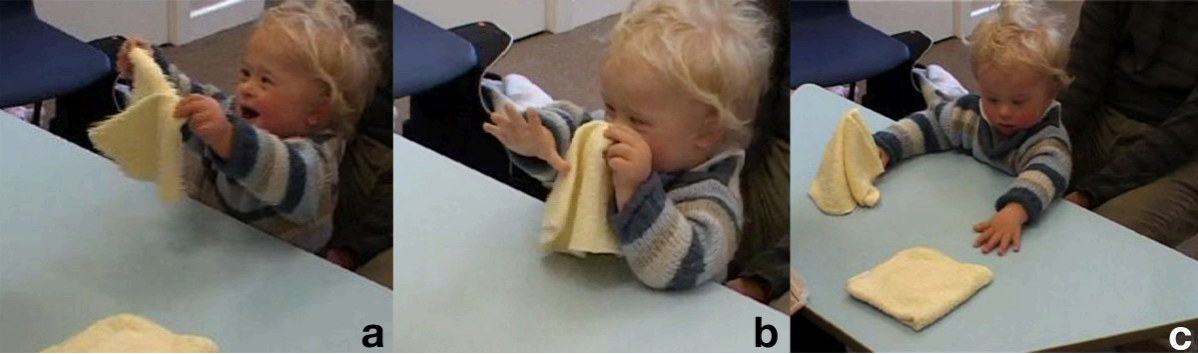		
Figure 6(a), Figure 6(b), Figure 6(c).		
34	Assess:	=↑Here it is! ((*lifts bracelet for K to grasp*))
35		(.2) ((*K takes bracelet*))
36	Assess:	YAE:::::*[*Y:= ((*clapping*))
37		*[*((*K smiles*)) **G. 4a**
38	Assess:	=You found *[*it!
39		*[*((*K looks at her and tighten both fists around the bracelet*))
40	Assess:	Very clever=*[*‘r you watching?
41		*[*((*A takes bracelet from K's hands*)) **G. 4b**
42	Assess:	.Hh I'm gonna h:ide it again= *[*you watching?
43		*[*((*A holds the bracelet up*))
44		.h hu:hh I'm gonna *[*hide it under he:re,
45		*[*((*A puts bracelet under left cloth, K looks there*)) **G. 4c**
46	Assess:	*[*you watching?
47		*[*((*A puts hands on cloths*))
48	Assess:	.Hs:*[*s::: oo:oo:::h ((*swaps the cloths*))
49		*[*((*K puts hands on the bracelet-cloth as it passes in front of him*)) **G. 5a**
50		.hhh °Where i:(h)s (h)i:t?° ((*breathy*))
51	Kevin:	Hu::: ((*reaches for both cloths, arms spread*)) **G. 5b**
52	Assess:	Where is the bra:celet? ((*smile voice*))
53	Kevin:	Ehhh ((*picks up the ‘wrong’ cloth and lifts it up*)) **G. 5c**
54	Assess:	Oh:h you like the cloths don't you?
55	Kevin:	UH::! ((*holds cloth up with both hands, moves it toward A, smiling*)) **G. 6a**
56		(.2)
57	Assess:	Yea:h. ((*unenthusiastic*))
58		(1.5)
59	Kevin:	Uhuhuhuh? ((*bow his face to touch the cloth*)) **G. 6b**
60		(6.0) ((*K looks at A, moves the cloth around and shakes it*,
61		*then reaches for the other one*)) **G. 6c**

Before reiterating the trial, the assessor models “success” by letting Kevin grab the bracelet, loudly praising him as he does that, and letting him hold and manipulate the toy for a few seconds (lines 34–39). She then announces she is going to hide the bracelet again, takes it from him and holds it up at his eye level before hiding it. The act of hiding the bracelet is also accompanied for its entire duration by utterances in a playful, breathy voice, typical of the expression of amazement or surprise; she keeps the same affective tone in the non-verbal vocalizations she utters while swapping the cloths around (line 48).

The continuous and affectively loaded voicing of the assessors is effective in keeping the child engaged, although by the same token she makes herself more salient. Kevin follows her gestures closely; as she slowly makes the cloth with the bracelet pass in front of him, he rests the fingertips of both hands on it (G. 5a). When she takes her hands off the cloths and addresses the child with the utterance °Where i:(h)s (h)i:t?°, one of his hands is still on the cloth with the bracelet, but he then vocalizes in response and stretches the other arm toward the left where the bracelet had been last seen (G. 5b). At this point he grabs the “wrong” cloth, lifts and inspects it, and extends it toward the assessor with a loud and seemingly expectant “UH::!” (line 51). What follows in conversational terms is akin to a dispreferred second assessment (Pomerantz, 1984): there is a gap after the end of the child's vocalization, then a “Yeah” markedly less loud then her previous one, ending with descending intonation and with no playful vibrancy to it. This low intensity reply, while taking up the child's utterance, withholds praise or acceptance for his act; it thus constitute implicit negative modeling by communicating to the child that the cloth is not what the game is about. In the six seconds of silence that follow, the assessor lets Kevin manipulate the cloth, until he drops it down and reaches for the other one. Conversationally, this act is akin to a repair, in which there is an attempt at redressing a previous exchange that had not achieved a positive completion (Schegloff et al., 1977).

While it seems that this time Kevin was more clearly trying to find the bracelet, we can also see that he attempted to pull at the cloth in the course of the swapping: he responded to the assessor sliding the cloth toward him as an invitation to grabbing (line 49), just as she had done with the bracelet at the beginning of this trial. In other words, Kevin might not be attending to the activity as a fixed hide-swap-find sequence in which he is supposed to act at the end, but rather as one having multiple entry points for him, in response to each assessor's move.

The task for the child is then to make up the rules as he goes along; the fact that there are rules and this is not free play is indexed by the reactions of the assessor to what he does. Despite, therefore, the Bayley's intent to assess “play skills,” the child's performance is geared toward making sense of the assessor's verbal and non-verbal conduct in relation to his actions. The type of play that is set up in the test is not auto-telic, i.e., is not independent from the interactional frame that first encourages the child to engage with the test material and then evaluates his actions or reiterates the task, but it is not “playing together” either, because the child is left alone at the point in which he has to demonstrate his “skill.” Each item administration resemble thus in format an instructional sequence (Marlaire and Maynard, 1990), in which a performance enacted upon request (like a school pupil's answering of a teacher's question) is followed by its evaluation, and where withholding of the follow up and/or the reiteration of the question can represent negative feedback.

At the end of the previous sequence, the assessor again commented on the child's lack of success, this time identifying his interest in the cloths as impeding a successful completion of the task (line 54). Before the subsequent trial (Extract 4), she does some more facilitating activities, but this time marks out the act of *uncovering* the bracelet from beneath the cloth, rather than the bracelet as such.

**Table:** **Extract 4** [Figures 7 and 8]

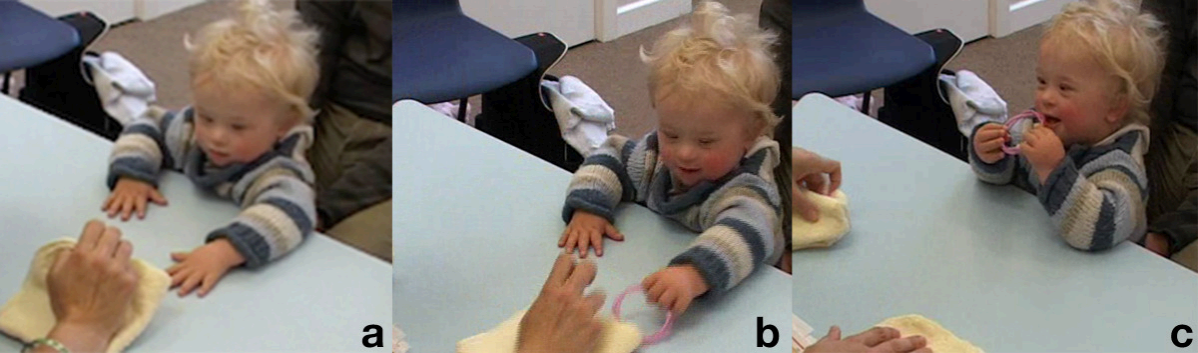		
Figure 7(a), Figure 7(b), Figure 7(c).		
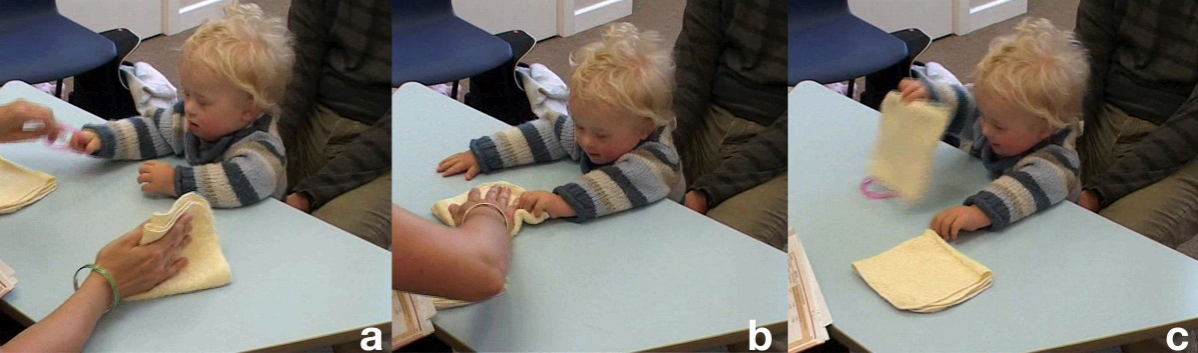		
Figure 8(a), Figure 8(b), Figure 8(c).		
62	Assess:	((*takes cloth [from K's hand*)),
63		*[>*°How about under this one=
64		*[=*ready?
65		*[*((*K pinches a corner of the cloth with the bracelet*))
66	Assess:	*[*.HHU!
67		*[*((*A lifts the corner up exposing the bracelet, K looks*)) **G. 7a**
68		(2.0) ((*A covers the bracelet again and looks at K*.))
69		((*K looks at the cloth without moving*))
70	Assess:	°Oh:h[h::: ((*lifts the cloth higher*))
71	Kevin:	[°Ehh:: °
72		(0.5)
73		° Look.° °there it is.° ((*A keeps cloth lifted up and slides the bracelet toward K*))
74	Kevin:	Eheeh ((*grabs the bracelet*)) **G 7b**
75	Assess:	YEAH:::! YOU'VE GO:T IT!
76		((*K smiles broadly, grasps the bracelet with fisted hands*)) **G. 7c**
77		HAHA:HA! VERY clever ri:ght.
78		(1.8) ((*A folds the cloths in four preparing for the next trial*))
79	Assess:	*[*Watchin' agai:n, (.) rea:dy::?
80		*[*((*Reaches over and seizes the bracelet, K keeps its grip*)) **G. 8a**
81		I'm gonna hide i:t, ((*pulls the bracelet toward her*))
82		under *[*here, you watching?
83		*[*((*taps bracelet on K's hand on the way to hiding it*))
84		(1.0) ((*A hides bracelet under the right cloth*))
85	Assess:	There it goe:s, hah go on.
86		*[*watchin=watchin=watchin=watchin=watchin,
87		*[*((*A switches positions of the cloths, bracelet goes to left hand side*))
88		*[*((*K grab the s edge of the bracelet-cloth and holds it*)) **G. 8b**
89	Assess:	.Hhhhhu (.) >where is i:t *[*go:ne?< ((*smile voice*))
90		*[*((*A takes hand off the cloth*))
91	Kevin:	(0.5) ((*K pinches then lifts the cloth hiding the bracelet*)) **G. 8c**
92	Assess:	*[*GOOD BO:Y VERY GOO:D=WELL DO:NE that's it.
93		*[*((*K holds the cloth up then puts it aside and takes the bracelet*))
94	Assess:	There it i:s?
95		(0.8)
96	Assess:	*[*VERY GOODH!
97		*[*((*K lifts the bracelet and stares at it*))
98		(1.0)
99	Assess:	Ready?=we've got o:ne mo:re to do::, ((*low mock voice*))

The assessor, before starting the trial proper, covers and uncovers the bracelet twice, first producing a continuous inhaling sound, the second time a shorter, louder and more marked aspirated sound, “.HHU!.” To this, the child stays still, while keeping the gaze on the cloth (G. 7a). The assessor then lifts the cloth up again, producing sounds, keeps it lifted and slides it forward so to invite Kevin to take hold of the bracelet. As he does it, she utters a loud, high pitch and smiling “YEAH:::!” then continues on with more words of praise in the same affective quality (line 77). In stark contrast with the child unresponsive attitude toward the bracelet in the first part of the sequence, the assessor with her reaction retrospectively constructs taking the bracelet as a highly positive and praiseworthy action, which in turn has the child displaying positive affect by smiling to her.

This did not count as success because the bracelet was in sight of Kevin when he took it; the assessor then goes on with the next trial. For the duration of the hiding and moving the cloths she keeps talking to Kevin, and taps the bracelet softly on his hand just before making it disappear under the cloth. The utterances are again delivered with a breathy, smiling voice, and those features become even more prominent when she addresses Kevin the question “.Hhhhhu (.) >where is i:t [go:ne?<.” The boy, who had kept the tips of his finger on the cloth with the bracelet while it was being swapped, lets go briefly of it just to grasp it with his other hand and lifts it up, finally completing the task. This provokes again a prolonged and loud praise by the assessor. As before, she lets him play with the bracelet for a short time; she then takes it away for the last trial (with the bracelet on the opposite side), which will be successful as well. This last sequence shows what seems a more single-minded approach to the task, which has the child keep track of the bracelet, lift the cloth as soon as it is free and then take the bracelet in his hand. Once, in other words, the expectations of the adult interactant were more transparent to him, he was able to mobilize a strategy to comply with them.

## Discussion

The analysis of the administration of the Bayley-III “Find the hidden object” showed two systems at work in the course of the test administration: the conversational organization, visible in the turn-taking exchange between assessor and child, and the play framework, activated by the quality of the assessor's communication and by the presence and use of the toys.

Both Henricks (2006) and Sicart (2014) stress that the experiential value of play is the connection it allows with other humans and the new possibilities it opens for that connection to be explored; our analysis aligns with those claims by illustrating how, if play is activated by an interactional partner, the relational aspect of the activity is foregrounded; once play is set off, the co-player becomes a source of cues for ways in which playing can develop. We have described the intent gaze of the child on the assessor, and his bit-by-bit reactions to her moves, showing that her conduct was for the child a very prominent component of the situation. We have seen the assessor sustaining the child's engagement by accompanying the physical actions required for the administration of the item with conversational moves with child-directed communication features, as well as play markers, thus investing the objects with interactional significance while inviting the child to act on them.

After the child's initial failure, the assessor relied on the established interactional frame for what we have described as *modeling*, i.e. selecting and marking out through intense positive affect parts of the successful sequence, i.e., first the grabbing of the bracelet then the uncovering of it. The child was seen to orient to those sequels of his actions until he was able to induce the more positive reaction consistently. Apparently, the hidden object to be found in this section of the test was the rule of the game itself, after which the child had enough resources to perform successfully. Without deciphering the verbal of the part communication (the “where is it”? of the assessor), what the child had at his disposal were the paraverbal features, such as intonation, volume and sound play; the assessor increased her use of them throughout the episode until the two of them seemingly reached a state of intersubjectivity about the matter at hand.

Maynard (2005) discusses interactional practices in terms of Gestalt configurations, and argues that, in the disembedded tasks used in tests, “local,” more detailed interpretations can prevail for children with difficulties over more “global” ones, which would identify the conventional type of action requested by their interactant. Local forms of interpretations are still rooted in ordinary interactional resources, but less likely to be used in the same context by children with typical development. Kevin thus might have been initially following a local move-countermove pattern, instead of responding to the full structure of the game, and only later, after some support, aligning with the task as proposed by this test item. On the other hand, we may be confronted with a specificity of this child, perhaps linked to Down syndrome, as the inclination to attend to the social component of a situation rather than the physical one, and tune in with the affective rather than semantic level of communication, as research in social skills observed in children with the condition may suggest.

We have also observed how the objects constituting the test item, expected to trigger certain behaviors on the basis of their physical characteristics, did not in fact suggest to the child a unique course of action. The cloths, designed to be neutral tools for hiding the bracelet to sight, were treated by him as having a variety of affordances, and appeared pleasant to manipulate, whereas the bracelet did not generate an immediate interest^6^. We know that the cloths had been introduced to replace the inconveniently heavy cups used in previous versions, and that any objects included or changed in the test were reviewed by panels of experts and pilot-tested (Albers and Grieve, 2007). Still, it may be difficult even for expert adults to predict the preferences of young children, even more so if the children have an atypical psycho-physical set-up. Furthermore, as discussed earlier, it is inherent in the nature of play to embed exploratory activities, and to allow improvised and innovative uses of objects. Children in an assessment situation, therefore, can find themselves involved in an activity that sports familiar features of play as they experience it at home or at the nursery, but where there are stricter constraints to the repertoires of actions that can be tried out. The assessor's behavior orients the child toward a right-or-wrong interpretation of his own actions, thus leaving the realm of play to enter that of instructional activities. This hybridization of activity frameworks might also be in the way of children's grasping the relevant level of response between local and global, as discussed above.

There may be more specific causes of confusion regarding the design of the activity. It has been argued that very young children understand the purpose of directives before being able to interpret what the directive is asking (Reddy, forthcoming). We have seen the child in this study responding regularly with both vocalizations and actions to the assessor's requests to find the bracelet, but without complying with the specific content of the request: if verbal comprehension is not fully present, the verbal part of the item administration may represent just a generic–and misleading–prompt for the child to act. As concerns requests of the “find the hidden object” type specifically, it has been observed that younger typical children, when the act of hiding is accompanied by verbal communication, do more “perseverative search errors”–i.e., searching for an object where it was last seen–than when there is no communication going with it, suggesting a systematic pragmatic misunderstanding of what the talk is doing in relation to the object ^7^ (Topál et al., 2008, cit, in Csibra and Gergely, 2009). These observations together suggest that the combination of verbal stimuli and object manipulation constituting “play” in assessments may put young and verbally delayed children onto a very different action trajectory compared to children with higher language competences.

In essence, we would like to argue that the way in which “play” unfolded in the interaction developing around the test item diverged substantially from the “play” that was embedded in this test item according to its designers. The assessor's competent direction in cueing the right acts is involuntary testimony to this discrepancy between a definition of play as a scripted manipulation of objects and one in which it is the product of interactional possibilities opened in a shared domain of action.

An obvious point is that the failure of a child to perform the required behavior cannot be unambiguously attributed to a deficit of the relative cognitive skills, and that the narrow range of actions allowed by the test, their imposed repetition, and the quick succession of different trials throughout the test are likely to trigger behavioral heuristics aimed at cutting down the task's repetition or conquering most praise. However, it is not our intention to undermine the Bayley III or psychological assessments in general^8^. Our interest lies in showing that extricating a child's performance from the bundles of interactional events happening in the course of a test administration can only be done by disciplinary practices with the power to retain selected features of a situation and erase the conditions of their coming into being. Such practices, that Foucault (1975) saw as the exertion of disciplinary authority, and that ethnomethodologists since Garfinkel (1967) have been out to discover (Housley and Fitzgerald, 2006) have the power to create “second nature,” (Gramsci, 1971; Pizza, 2012), namely to represent socially produced human traits and qualities as originated by natural causes. Societal and clinical understanding of conditions such as the Down syndrome are largely constituted via specialized instruments, like the Bayley-III, that set definitions and boundaries of adequate performance, limiting the knowledge that can derive from letting diversity have its way.

## Conclusions

The study illustrated that play in assessment loses some of the core features of play, such as free exploration and novel uses of objects, and instead, being structured as small single tasks ending with implicit or explicit evaluations, borders with verification-type instructional activities. The range of play skills the assessment aims at measuring is therefore limited to a diminutive version of “play.”

The study also suggests that a better understanding of the way verbal communication and object manipulation combine during the administration of test items could be beneficial in the design and evaluation of tasks: assessments designed to be language-free but which nonetheless involve language as a support to engagement can become very different entities from what the test designer might have envisioned. In such cases, it is not standardization that would help determine what skills come into play, but rather the systematic microanalysis of actual episodes of test administrations, in order to reconstruct the sense-making procedures occurring between the interactants in relation to the tasks.

As concerns the choices made in this study, while focusing on a single episode was instrumental to follow closely the trajectory from failure to success, and unpick the moment- by-moment procedures supporting mutual understanding, it could have also been informative to examine the whole assessment and explore the types of interactional trajectories set out by different types of tasks. A promising path of investigation would be to follow the same children at home and in different institutional settings, for a more robust interpretation of the kind of resources a child is drawing from, and to ascertain whether the competences developed in familiar settings are transferred across when children find themselves in testing situations.

## Ethics statement

An ethics review had been carried out at the time of the initial recording following regulations of the Down Syndrome Educational International Trust, with full consent obtained from parents. Parents of the six children involved in the second study using the same recordings, done by the second author of this paper for her BSc dissertation, were contacted again, and asked to renew their consent; the ethics application was reviewed by the Ethical Committee of the Department of Psychology of the University of Portsmouth UK, which follows the guidelines of the British Psychological Society. Finally, the mother of the only child who is discussed in this paper has been contacted to clear permission on the uses of frame grabs from the video. The mother has been sent the grabs, and has provided written consent to their use. The written informed consents are in accordance with the Declaration of Helsinki.

## Author contributions

The paper has been mainly written by AF; JS is responsible for the acquisition and selection of data as well as for a large input in the analysis. SB has provided access to the data and has given a substantial contribution concerning the Bayley Assessment and Down syndrome education and intervention.

### Conflict of interest statement

The authors declare that the research was conducted in the absence of any commercial or financial relationships that could be construed as a potential conflict of interest.

## Footnotes

^1^The notion of frame was later elaborated by Goffman (1974) into an encompassing theory of communication.

^2^The delay is established according to following criteria: 25% delay in functioning when compared to same age peers, standard deviation units below the mean of a reference group and performing a certain number of months below chronological age (Maccow, 2008).

^3^Goodwin defines highlighting “a way of re-organizing a domain of scrutiny in terms of the tasks of the moment” (2003, p.245).

^4^This comment is addressed to the father despite the second person pronoun is indexing the child, and the father laughs. It is common for test administrators to take care of the parents' accountability about their child's performance.

^5^True, the assessor asked verbally to find the bracelet, however the verbal instruction is only accessory to the test, which is supposed to work independently of the language instructions.

^6^Shukla (2010) reports that it was common for the children she observed to show interest in the cloths during ‘Find the hidden object.

^7^Csibra and Gergely (2009:152) argue that the child could see “the experimenter's hiding actions as a communicative demonstration of some generalizable information (e.g. that container A is ‘for’ storing the kind of objects being hidden) rather than an interactive hiding–finding game.”

^8^As for the Bayley III in particular, there are indications that, in correcting bias identified for the previous versions, it may now in fact overestimate children's capabilities (Johnson et al., 2014).

## Appendix

### Transcription symbols

The transcription used in this paper is the standard for Conversation Analysis and is based on Jefferson (2004a).

**Table:**

Mea::t	Colon(s): Extended or stretched sound.
Fresh	Underline: Emphasis.
(.)	Micropause, pause of less than (0.2).
(1.2)	Timed Pause: Intervals occurring within and between same or different speaker's utterances in tenths of seconds.
(())	Double Parentheses: Contextual information.
() (we're)	Single Parentheses: non hearable speech (empty) or uncertain interpretation (with words)
Yeah.	Period: Falling vocal pitch.
Yeah?	Question mark: Rising vocal pitch.
Yeah!	Exclamation mark: animated tone.
HE DID	Caps: Marked loudness compared to surrounding talk.
[	Square bracket: Marks the beginning point at which current talk is overlapped by another speaker's talk.
*[*	Bracket in italics mark simultaneous onset of movements or gestures with talk of same or other speaker
↓↑	Pitch resets; marked rising and falling shifts in intonation.
=	Latching of contiguous utterances, fast succession of the spates of talk united by =
°Well	A passage of talk noticeably softer than surrounding talk.
> <, < >	Less Than/Greater Than Signs: Portions of an utterance delivered at a pace noticeably quicker (> <) or slower than surrounding talk.
But-	Hyphen: Halting, abrupt cut off of sound or word.
.hhh	Single or multiple ‘h’ letter preceded by dot: audible inbreaths.
h (h)	Single or multiple ‘h’ letter alone or in brackets: audible stand alone outbreaths, sighing; within words: breath emission through (like laughter or pfh-ing).
